# A retrospective descriptive review of community-engaged research projects addressing rural health priorities

**DOI:** 10.1186/s12909-024-05791-7

**Published:** 2024-07-29

**Authors:** Bushra Farah Nasir, Bruce Chater, Matthew McGrail, Srinivas Kondalsamy-Chennakesavan

**Affiliations:** 1 Mayne Academy of Rural and Remote Medicine, Rural and Remote Medicine Clinical Unit, Medical School, Faculty of Medicine, The University of Queensland, Theodore 4719, QLD Australia; 2 Toowoomba Regional Clinical Unit, Medical School, Faculty of Medicine, The University of Queensland Toowoomba, 6 Range Street, Mount Lofty, Toowoomba, QLD 4350 Australia; 3 Rockhampton Regional Clinical Unit Medical School, Faculty of Medicine, The University of Queensland, Rockhampton 4700, QLD Australia

**Keywords:** Rural health, Rural medicine, Medical education, Community engagement

## Abstract

**Background:**

Most rural populations experience significant health disadvantage. Community-engaged research can facilitate research activities towards addressing health issues of priority to local communities. Connecting scholars with community based frontline practices that are addressing local health and medical needs helps establish a robust pipeline for research that can inform gaps in health provision. Rural Health Projects (RHPs) are conducted as part of the Doctor of Medicine program at the University of Queensland. This study aims to describe the geographic coverage of RHPs, the health topic areas covered and the different types of RHP research activities conducted. It also provides meaningful insight of the health priorities for local rural communities in Queensland, Australia.

**Methods:**

This study conducted a retrospective review of RHPs conducted between 2011 and 2021 in rural and remote Australian communities. Descriptive analyses were used to describe RHP locations by their geographical classification and disease/research categorisation using the International Classification of Diseases and Related Health Problems – 10th Revision (ICD-10) codes and the Human Research Classification System (HRCS) categories.

**Results:**

There were a total of 2806 eligible RHPs conducted between 2011 and 2021, predominantly in Queensland (*n* = 2728, 97·2%). These were mostly conducted in small rural towns (under 5,000 population, *n* = 1044, 37·2%) or other rural towns up to 15,000 population (*n* = 842, 30·0%). Projects mostly addressed individual care needs (*n* = 1233, 43·9%) according to HRCS categories, or were related to factors influencing health status and contact with health services (*n* = 1012, 36·1%) according to ICD-10 classification.

**Conclusions:**

Conducting community focused RHPs demonstrates a valuable method to address community-specific rural health priorities by engaging medical students in research projects while simultaneously enhancing their research skills.

## Background

People living in rural regions experience a greater burden of health disparities and disadvantages across most health and wellbeing domains [1, 2]. Despite clear inequities existing between rural and urban populations, there have been limited research-based strategies focused on addressing community-level health and medical priorities [3]. To achieve a better understanding of health issues impacting rural communities, innovative research to identify the health issues directly impacting people living in rural areas can result in community-focused strategies to address these challenges.

In Australia, immense inequities in research funding targeting rural health strongly diminish the capacity for rural health research supported by an integrated academic infrastructure [4]. A large portion of the research being conducted in rural communities depends upon busy clinician researchers, who work within the local health and medical workforce [5]. Improving sustainability of rural focused researchers and clinician academics thus requires a focused approach to providing critical skills development and community-centred research opportunities that are integrated within the medical curriculum. Rural and remote research involves high levels of community engagement, rural-based immersion opportunities and positive learning experiences that result in ‘socially accountable’ research activities [5]. A tailored, community-engaged approach also significantly impacts future rural practice intent [6, 7], which is a critical government agenda that aims to sustain a rural workforce that is committed to work in underserved rural communities. For anyone intending to practice in a rural or remote location, the importance of developing research and analytical skills is more significant, given the complex nature of rural environments [6].

Preparing medical students for a rural career in evidence-based medicine requires sufficient research training and experiences to develop both their ability to appraise clinical evidence and their analytical skills required in medical practice [8]. A recent review of Australian medical students confirmed that the inclusion of scholarly activities to support the development of basic research skills and critical evaluation is not universally embedded within medical degree programs [9, 10]. Similarly, a study exploring attitudes and participation in research activities by medical students in Australia found that only 45% of the 704 survey respondents had participated in a research project [11]. To instil scholarly research skills development, the University of Queensland (UQ) in Australia incorporates various units that are aimed to develop research skills as part of their medical training. In year three of the four-year MD program, all domestic students undertake a Rural and Remote Medicine (RRM) placement under the Mayne Academy of Rural and Remote Medicine clinical unit. Alongside clinical teaching and training, a Rural Health Project (RHP) forms part of the RRM placement during which students complete a small research project with an emphasis on identifying and addressing local community priorities.

The RHPs are developed through a local iterative process that balances the needs of the rural communities, the advice of the locally based supervisors, and student skills and interests, using the community-engaged research conceptual framework principles [12]. RHPs are conducted within rural hospitals, general/family practice, or a combination of both, as well as some projects being undertaken within the community but outside of a clinical setting. An example is that of a former mining engineer doing medicine arranged an underground gold mine rescue scenario that was filmed as part of the RHP. The video was used for training purposes, providing an output beneficial to the local community. As a result, students hone their research skills and involve themselves in multidisciplinary practice and participatory research in the context and culture of a rural community.

The RHP is integrated with the flow of phase one pre-clinical programs and fits in with other RRM assessments and practical experiences. They are designed to be carried out within a Quality Improvement framework that aims to develop an understanding of rural health service delivery, while learning to work collaboratively in gaining an understanding of health status and issues of priority for local rural communities in which the students are placed. The RHP pedagogical approach is underpinned by a sociocultural theory [13–15]. Students work under interactive guidance and supervision regarding the cognitive and experiential aspects of their activities, with intensive immersion in the tasks being carried out, relying on self-motivation, initiative and problem-solving. During the RHPs, students learn how to critically analyse a clinical topic, engage with community members and clinicians, and collaborate as required. Students are also responsible for planning and conduct of the project and producing practical resources or an end-product that is then presented in a written academic report. The key elements of the RHPs are to harness the opportunity of placement at a rural site by identifying a health service need or locally relevant knowledge gap to be addressed in consultation and engagement with the community.

More than 270 RHPs are conducted every year within UQ as part of the RRM unit spread over 50 smaller rural and remote communities. The overarching goal for each student’s RHP is to develop a long-term, solution-orientated plan of benefit to the local community.

This study aimed to describe the geographic coverage of RHPs, the health topic areas covered and the different types of RHP research activities conducted. It also provides meaningful insight of the health priorities for local rural communities in Queensland, Australia.

## Methods

This study is a retrospective analysis of all RHPs conducted by medical students as part of their RRM unit, during Year 3 of their medical training at UQ. Specific data available for each RHP were the project title, the year it was conducted, and the location, each of which was collected as part of standard administrative procedures by the RHP coordinators. No identifying information about the students were collected, thus no other linkage was possible such as to student characteristics. Each RHP is conducted by one medical student.

Location information was coded by the researchers (BN, SKC, MM) using the Modified Monash Model [16] categories. Using descriptive information from the RHP title, researchers also coded the RHPs using the International Classification of Diseases and Related Health Problems – 10th Revision (ICD-10) codes and the Human Research Classification System (HRCS) categories. The primary researchers involved in data setup (SKC and BN) conducted the categorisation and coding of the data, followed by a researcher (MM) reviewing and confirming accurate categorisation and coding. A descriptive analysis of the RHPs was conducted to explore ICD-10 codes and HRCS categories according to rural, remote, and regional locations using the Modified Monash Model (MMM) [16] Classification system.

## Results

A total of 2974 projects were reviewed in this study. After coding and removing projects with missing key information, and projects that were conducted outside of Australia, a total of 2806 RHPs remained.

The distribution of RHPs within each state based on regional location is described in Table 1. A majority of RHPs were conducted in Queensland (*n* = 2728, 97·2%). Due to the small number of RHPs within Victoria, New South Wales, the Northern Territory and Western Australia, these states were combined into a single category (other). According to the MMM categories, most RHPs were conducted in small rural towns (MMM-5, *n* = 1044, 37·2%), or medium rural towns (MMM-4, *n* = 842, 30·0%). Additionally, nearly 17% of RHPs were conducted in Australia’s remote areas (MMM-6 and MMM-7, *n* = 468). A small number of projects (*n* = 195, 7·0%) were conducted in areas not targeted under the RRM program (MMM-1 and MMM-2). These RHP locations were used by students mainly because of administration related factors, including students not being able to travel to a suitable location during COVID-19 related restrictions.

**Table 1:** Rural Health projects distributed by regional classifications and states in Australia. *MMM: modified Monash Model categories

MMM*	QLD	Other	Total
**MMM-1 (Metropolitan)**	24 (96·0)	1 (4·0)	25 (0·9)
**MMM-2 (Regional city)**	157 (92·4)	13 (7·6)	170 (6·1)
**MMM-3 (Large rural)**	254 (98·8)	3 (1·2)	257 (9·2)
**MMM-4 (Medium rural)**	837 (99·4)	5 (0·6)	842 (30·0)
**MMM-5 (Small rural)**	1042 (99·8)	2 (0·2)	1044 (37·2)
**MMM-6 (Remote)**	173 (83·6)	34 (16·4)	207 (7·4)
**MMM-7 (Very remote)**	241 (92·3)	20 (1·7)	261 (9·3)
**Total**	2728 (97·2)	78 (3.0)	2806 (100·0)

The frequency of the RHPs according to HRCS categories, and the ICD-10 codes are illustrated in Tables 2 and 3 respectively. Analysis was limited to each HRCS category or ICD-10 code having at least 20 RHPs. The most frequent MMM category within each HRCS category and ICD-10 code illustrate the regional distribution within each research topic area. According to the HRCS categories, RHPs most frequently addressed *Individual care needs* (*n* = 1233, 43·9%) and were conducted in MMM-5 locations (*n* = 487, 37·1%). Similarly, according to the ICD-10 codes, RHPs most frequently explored *Factors influencing health status and contact with health services* (*n* = 1012, 36·1%) and were conducted in MMM-5 locations (*n* = 347, 34·2%).

**Table 2:** Rural Health projects categorised according to the human research classification system categories

HRCS Research Activity Category	*n* (%)	*n* (%) within top MMM	MMM
Individual care needs	1233 (43·9)	487 (37·1)	MMM-5 (small rural)
Organisation and delivery of services	634 (22·6)	208 (32·8)	MMM-5 (small rural)
Management and decision making	457 (16·3)	153 (33·5)	MMM-4 (medium rural)
Primary prevention interventions to modify behaviours or promote well-being	229 (8·2)	97 (42·4)	MMM-5 (small rural)
Vaccines	66 (2·4)	26 (39·4)	MMM-5 (small rural)
Population screening	64 (2·3)	27 (42·2)	MMM-5 (small rural)
Nutrition and chemoprevention	42 (1·5)	19 (45·2)	MMM-5 (small rural)
Psychological and behavioural	25 (0·9)	14 (56·0)	MMM-5 (small rural)
End of life care	25 (0·9)	10 (40·0)	MMM-4 (medium rural)
Other	31 (1·1)	n/a	n/a

**Table 3:** Rural Health projects categorised according to the International Statistical Classification of Diseases and related health problems – 10th revision (ICD-10) codes

ICD-10 codes	*n* (%)	*n* (%) within top MMM	MMM
Factors influencing health status and contact with health services	1012 (36·1)	347 (34·2)	MMM-5 (small rural)
Mental, and behavioral and neurodevelopmental disorders	337 (12·0)	162 (48·1)	MMM-5 (small rural)
Endocrine, nutritional, and metabolic diseases	271 (9·7)	124 (45·7)	MMM-5 (small rural)
Certain infectious and parasitic diseases	171 (6·1)	55 (32·2)	MMM-5 (small rural)
Neoplasms	171 (6·1)	72 (42·1)	MMM-5 (small rural)
Diseases of the circulatory system	133 (4·7)	40 (30·1)	MMM-5 (small rural)
Injury, poisoning, and certain other consequences of external causes	106 (3·8)	40 (37·7)	MMM-4 (medium rural)
Symptoms, signs, and abnormal clinical and laboratory findings, not elsewhere classified	95 (3·4)	36 (37·9)	MMM-4 (medium rural)
Diseases of the respiratory system	89 (3·2)	34 (38·2)	MMM-5 (small rural)
Pregnancy, childbirth, and the puerperium	84 (3·0)	38 (45·2)	MMM-4 (medium rural)
Diseases of the genitourinary system	66 (2·4)	28 (42·4)	MMM-5 (small rural)
Diseases of the musculoskeletal system	60 (2·1)	23 (38·3)	MMM-5 (small rural)
Diseases of the skin and subcutaneous tissue	50 (1·8)	18 (36·0)	MMM-4 (medium rural)
Diseases of the digestive system	47 (1·7)	15 (31·9)	MMM-5 (small rural)
External causes of morbidity and mortality	39 (1·4)	16 (41·0)	MMM-5 (small rural)
Diseases of the eye and adnexa	27 (1·0)	9 (33·3)	MMM-5 (small rural)
Other	48 (1·7)	n/a	n/a

Examples of RHPs conducted in HRCS Research Activity codes and ICD-10 codes (Table 4) highlight some of the key health research topics that the RHPs have addressed.

**Table 4:** Rural Health Project examples from the Human Research Classification System Categories and the International Statistical Classification of Diseases and related health problems – 10th revision (ICD-10) categories

HRCS Research Category	Rural Health Project Examples
Individual care needs	• *Diabetes - health promotion in adolescents* • *Enhancing patient engagement in chronic disease*
Organisation and delivery of services	• *Telehealth in Beaudesert General Practice and Skin Cancer Clinic* • *Closing the Gap – Practice Incentives Program: Indigenous health incentives*,* Gladstone*
Management and decision making	• *Burn-out in healthcare workers in the rural setting* • *Antenatal exercise and gestational diabetes management at Kingaroy Hospital*
Primary prevention interventions to modify behaviours or promote well-being	• *Education and recognition of depression within a rural mining town* • *Interventions for bariatric surgical follow-up in rural Queensland*
Vaccines	• *The Q-fever vaccine: education and awareness in Woorabinda* • *Covid-19 - An educational incentive*
**ICD-10 Codes**	
Factors influencing health status and contact with health services	• *A comparison of two new tools to communicate the triage process to reduce patient dissatisfaction with the waiting time at Roma Hospital emergency department.* • *Interhospital transfers from Roma Hospital*
Mental, and behavioural and neurodevelopmental disorders	• *Health in the rural men’s shed: depression.* • *Mental health: measuring baseline knowledge and efficacy of an educational presentation in rural adolescents*
Endocrine, nutritional, and metabolic diseases	• *Exercise as medicine in the Bundaberg region’s veteran population* • *Assessing the viability of opportunistic screening for undiagnosed Diabetes and pre-Diabetes in a busy rural emergency department*
Certain infectious and parasitic diseases	• *Meningococcal W outbreak – The central Australian context* • *Improving Access to Tetanus Prophylaxis in the Remote Community of Queensland*
Neoplasms	• *The exposed population - risk factors for skin malignancy* • *Sunspot hot spot - Evaluating and improving skin cancer detection and prevention strategies*

## Discussion

This study demonstrates the approach of immersive rural health research projects, conducted as part of medical curriculum in Australia. They describe how research activities conducted within rural communities can help address rural health priorities specific to each community, while also providing a practical approach for medical students to become involved in community-engaged research projects. The review also highlights the diverse nature of RHP topics that are community-identified issues relevant to the local communities. Communities undertake a collaborative process with the supervisor and student, to identify areas of focus that meets their needs. The resulting research activities conducted as part of the RHPs provide practical resources for immediate translation or direct evidence to support future interventions targeting improved rural health outcomes. A similar but smaller scale research initiative in Australia highlights that as part of a graduate medical program conducted during a 12-month GP placement in a rural, regional, or remote community in New South Wales, an increased understanding of local health issues in regional, rural and remote communities, and increased engagement with and acceptance of medical students in these communities was seen [17].

Unsurprisingly given that they are part of the UQ curriculum, most RHPs were conducted within Queensland. These were most commonly situated within small and medium sized rural towns and/or inner-regional locations, focused on Individual care needs. The HRCS category addressing Individual care needs explores several aspects of patients and service user care needs including quality of life, management of symptoms, disease management, prevention, and health service needs [18]. These issues correlate with multiple reports that continue to highlight the ongoing issue of access to primary health care services and higher levels of disease that impacts health outcomes within rural locations [19–21]. Similarly, according to the ICD-10 codes, RHPs most commonly explored factors influencing health status and contact with health services. Additionally, factors influencing primary health care access and the service needs of rural and remote communities is an ongoing concern [20]. The category of mental, behavioural, and neurodevelopment disorders was the second highest coded research project, highlighting its importance to these communities. A 2019 report by the Royal Australian College of General Practitioners corresponds with this finding, as it reported psychological issues as the most commonly managed health issue by General Practitioners (65%) [20].

Literature acknowledges challenges surrounding research activity during medical education. Time constraints (*n* = 460; 65·3%) and uncertainty surrounding how to find research opportunities (*n* = 449; 63·8%) are common barriers to research [11]. Other studies also highlight the lack of time (77·4%), and lack of formal research activity within the curriculum (76%), as well as lack of mentorship (70·1%) [22]. Solutions include protected research time, financial and other academic support that would help facilitate and improve participation in research projects [23]. By providing an integrated research project that is assessed and embedded within the medical curriculum of the MD degree, this study highlights how these challenges can potentially be mitigated. The importance of providing medical students the opportunity to learn and conduct research during their medical education is essential to prepare future rural clinician researchers [10].

A significant strength of this study is the diversity and volume of rural health projects conducted. Additionally, a greater understanding of the health priorities were identified for rural communities. The strength of this study also highlights the number of successfully completed RHPs, whereby students gained valuable advantage to understand the process of gathering and synthesising data and developing important outcomes or resources relevant to their rural placement communities. There are however several limitations to this study. Although the ICD-10 and HRCS coding systems can categorise medical health related research activity, they are limited in their design to adequately classify rural health research projects relating to geographical factors. This limitation may restrict the generalisability of findings from this study. Another limitation is that this study relied on administrative data, which did not include other valuable information such as student characteristics or placement contexts within each of the locations. Additionally, the outcomes of each RHP were also not available. The categorisation process was also based on the understanding of the researchers, however, to overcome this bias, a systematic approach to categorisation was used, whereby all researchers checked and verified consensus on the categorisation of each RHP.

## Conclusion

The integration of research projects focused on both understanding rural health disadvantages and suitable interventions as part of a medical students training and learning experience is an innovative method to address rural health challenges, while encouraging medical students to enhance their research skills. Students address topics of local priority through their RHPs, increase their involvement with the rural communities and other health professionals and develop an increased understanding of local health issues in rural and remote communities. Furthermore, advancing opportunities to undertake integrated rural health research activities within a medical student’s degree can progress a student’s scholarship, encouraging future academic endeavours. Such community-engaged, locally based rural health projects also allow us to better understand the unique factors associated with health and health care within rural communities, as well as the underlying factors explaining rural versus urban differences. These research focused activities ultimately not only benefit the local communities in which such projects are conducted, but also provide an educational model that achieves academic outcomes benefitting the medical student.

## Data Availability

The datasets used and/or analysed during the current study available from the corresponding author on reasonable request.
